# Association between COVID‐19 and myasthenia gravis (MG): A genetic correlation and Mendelian randomization study

**DOI:** 10.1002/brb3.3239

**Published:** 2023-08-28

**Authors:** Dongren Sun, Liangdan Tu, Xiaofei Wang, Qin Du, Rui Wang, Ziyan Shi, Hongxi Chen, Hongyu Zhou

**Affiliations:** ^1^ Department of Neurology West China Hospital, Sichuan University Chengdu Sichuan China

**Keywords:** causal effect, COVID‐19, Mendelian randomization, myasthenia gravis

## Abstract

**Background:**

Observational studies have suggested an association between coronavirus disease 2019 (COVID‐19) and myasthenia gravis (MG). Here, we aimed to estimate the genetic correlation and causal relationship between COVID‐19 susceptibility, hospitalization, severity, and MG phenotypes using linkage disequilibrium score regression (LDSC) and Mendelian randomization (MR) approach.

**Methods:**

Summary statistics of COVID‐19 susceptibility, hospitalization, and severity were used as instrumental variables for exposure traits. Large‐scale genome‐wide association study (GWAS) data for MG were used as outcome traits. The inverse variance weighted approach was used for the main MR analysis, complemented by MR‐Egger, weighted median, simple mode, and weighted mode methods. Sensitivity analysis was implemented using Cochran's *Q* test, MR‐PRESSO method, and MR‐Egger intercept test.

**Results:**

LDSC analysis did not reveal any genetic correlation among COVID‐19 susceptibility, hospitalization, severity, and MG phenotypes, including MG, early‐onset MG, and late‐onset MG (*p* > .05). Our MR analysis did not provide evidence supporting a causal effect of COVID‐19 susceptibility, hospitalization, or severity on MG phenotypes (*p* > .05). Extensive sensitivity analysis strengthened the robustness and consistency of the MR estimates.

**Conclusion:**

Our study did not find evidence of a genetic correlation or causal relationship among COVID‐19 susceptibility, hospitalization, severity, and MG. Future studies with more GWAS data are needed to evaluate the association between COVID‐19 phenotypes and MG and its subgroups.

## Graphical abstract

No genetic correlation or causal relationship between COVID‐19 susceptibility, hospitalization, severity, and myasthenia gravis.

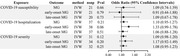

## 1 INTRODUCTION

Coronavirus disease 2019 (COVID‐19), caused by severe acute respiratory syndrome coronavirus 2 (SARS‐CoV‐2), has triggered a global panic and posed a substantial public health threat (Taquet et al., 2022). Besides its impact on the respiratory system, SARS‐CoV‐2 can invade the nervous system, resulting in a wide range of neurological symptoms (Aghagoli et al., 2021). SARS‐CoV‐2 infection can elevate inflammatory proteins, induce immune responses involving T and B cells through interferon, trigger the formation of a “cytokine storm,” lead to abnormal immune responses, alter the function of immune cells, and initiate the development of neuroimmune diseases (Amruta et al., 2021; Ismail & Salama, 2022; Payus et al., 2022; Singh et al., 2021).

Recently, several case reports and series have emerged, documenting cases of myasthenia gravis (MG) developing after COVID‐19 infection or vaccination (Chavez & Pougnier, 2021; Fanella et al., 2022; Restivo et al., 2020; Sriwastava et al., 2021). The temporal relationship observed in these cases has led to speculation about a possible association between COVID‐19 and MG (Shah et al., 2022). Emerging evidence suggests that COVID‐19 may aggravate MG symptoms or increase the risk of developing MG (Alcantara et al., 2023). A population‐based matched cohort study showed that adults with MG who contracted SARS‐CoV‐2 had a higher risk of hospitalization and mortality compared with controls (Alcantara et al., 2023). Another small study found increased mortality and incidence rates among MG patients compared to the general population of COVID‐19 patients (Muppidi et al., 2020). However, other studies have failed to find an increased risk of MG associated with COVID‐19. For example, a cross‐sectional study found no effect of COVID‐19 on MG progression (Businaro et al., 2021). Furthermore, a 10‐year survey of real‐world data from Germany found no evidence of higher MG incidence or hospitalization rates in 2020 compared to pre‐pandemic years, although this observation may have been influenced by the COVID‐19 pandemic (Wartmann et al., 2023). In addition, the safety of SARS‐CoV‐2 vaccination in MG patients remains controversial. Considering COVID‐19 vaccines as attenuated forms of the virus, investigating the potential association between COVID‐19 and an increased risk of MG may provide new insights.

Additionally, it is crucial to acknowledge the limitations of observational studies, which can be susceptible to confounding factors, reverse causality, and biases and only establish correlations rather than causal effects. Consequently, it is imperative to employ other research methods to elucidate the potential association between COVID‐19 and MG.

With the increasing availability of genome‐wide association study (GWAS) data, researchers can use linkage disequilibrium score regression (LDSC) and Mendelian randomization (MR) to gain further insight into the relationship between COVID‐19 and MG at the genetic level (Bulik‐Sullivan et al., 2015). These approaches provide valuable, practical, ethical, and cost‐effective alternatives. LDSC is a widely used method that helps to identify the common genetic structure of complex traits in humans, estimate the genetic power of diseases, and test their genetic correlations while accounting for true polygenicity and confounding biases in GWAS (Bulik‐Sullivan et al., 2015). MR analysis takes the advantage of genetic variants as instrumental variables (IVs) guided by Mendelian laws to assess the association between exposure and outcome using summary data from GWAS, ultimately simulating a natural randomized controlled trial (Smith & Ebrahim, 2003; Sun et al., 2023). Through MR, researchers can effectively minimize residual confounding and reverse causation issues (Yuan & Larsson, 2022), which are inherent limitations of observational studies. The MR method has been successfully applied to identify reliable risk factors associated with various diseases, including COVID‐19 and MG (Peng et al., 2022; Ponsford et al., 2020; Zhong et al., 2022).

Given that the relationship between COVID‐19 and MG is not clear, we used recent large‐scale GWAS summary data on COVID‐19 susceptibility, hospitalization, severity, and MG phenotypes, including all MG, early‐onset MG, and late‐onset MG, combined with LDSC and MR methods to evaluate their genetic associations and causal relationships.

## 2 METHODS

### 2.1 Data sources

The summary level data for COVID‐19 phenotypes (R7) were obtained from the COVID‐19 Host Genetics Initiative, including COVID‐19 susceptibility (159,840 cases vs. 2782,977 controls), hospitalization (44,986 cases vs. 2,356,386 controls), and severity (18,152 cases vs. 1145,546 controls) (COVID‐19 Host Genetics Initiative, 2020). Specifically, COVID‐19 susceptibility was defined based on a positive SARS‐CoV‐2 infection (e.g., RNA RT‐PCR or serology test), electronic health record evidence of SARS‐CoV‐2 infection (using International Classification of Diseases or physician notes), or self‐reported infections from the patients. COVID‐19 hospitalization referred to hospitalized patients with COVID‐19, whereas COVID‐19 severity represented COVID‐19 hospitalized patients who died or required mechanical ventilation support. Previous studies have presented more detailed descriptions (Butler‐Laporte et al., 2021; Leong et al., 2021). The summary data for MG were obtained from the largest recently published GWAS study, which included all MG (1873 cases vs. 36,370 controls), early‐onset MG (595 cases vs. 2718 controls), and late‐onset MG (1278 cases vs. 33,652 controls) (Chia et al., 2022). Early‐onset MG was defined as patients with age 40 or younger, whereas late‐onset MG was defined as patients with ages above 40. It is worth noting that since acetylcholine receptor antibody‐positive (AchR+) is found in 90% of generalized MG patients, this GWAS study only focused on these antibodies and did not investigate patients with muscle‐specific kinase (MuSK) or lipoprotein‐related protein 4 (LRP4) antibodies.

### 2.2 Genetic correlation analysis

LDSC is a robust and powerful tool used for analyzing genetic correlations between complex diseases and traits. This method involves regression analysis on the chi‐squared statistics derived from GWAS data for one phenotype or the cross‐product of chi‐squared statistics for two phenotypes (Bulik‐Sullivan et al., 2015; Kappelmann et al., 2021). Cross‐trait LDSC is an extension of single‐trait LDSC. Although sample overlap can affect the intercept of the regression model, it does not affect the slope, so the estimation of genetic correlation remains robust in the presence of sample overlap (Yang et al., 2022). In our study, we utilized LDSC (v1.0.1, available at https://github.com/bulik/ldsc) to conduct cross‐trait LDSC analysis. Specifically, we examined the genetic correlation among COVID‐19 susceptibility, hospitalization, severity, and MG using the regression slope derived from the LDSC analysis.

### 2.3 Mendelian randomization analyses

In this study, we used genetic variations associated with exposure as IVs to examine the potential causal relationship between exposure and outcomes using MR approach. The primary MR estimation method used was the multiplicative random effects inverse variance weighted (IVW) method, supplemented by MR‐Egger, weighted median, simple mode, and weighted mode methods. The IVs used in our analysis were genome‐wide statistically significant single nucleotide polymorphisms (SNPs) with a significance threshold of *p* < 5 × 10^−8^, within a range of 10,000 kb, and with low linkage disequilibrium (*r* ^2^ < .001) and strong IV strength (*F* statistic > 10) (Cai et al., 2021). To quantify the genetic heritability of individual COVID‐19 phenotypes, such as susceptibility, hospitalization, and severity, on different subtypes of MG, we used the formula 2 × BETA^2^ × (1 − EAF) × EAF, where BETA and EAF represent the genetic estimate strength and the effect allele frequency of the COVID‐19 phenotype IVs (Burgess & Thompson, 2011; Pierce et al., 2011). To assess the validity of our MR estimates, we monitored heterogeneity using Cochran's *Q* test and investigated horizontal pleiotropy using the MR‐Egger intercept test and MR Pleiotropy Residual Sum and Outlier (MR‐PRESSO) method. The mRnd online platform was used to calculate the statistical power of MR analysis for assessing the impact of COVID‐19 susceptibility, hospitalization, and severity on MG phenotypes (Brion et al., 2013). Analysis with a power greater than .8 was considered sufficient. In addition, we performed a reanalysis by screening SNPs associated with potential confounders, including age, thymoma, and autoimmune diseases (Alqarni et al., 2021; Wang et al., 2017) using the PhenoScanner tool (Kamat et al., 2019). Statistical significance was defined as *p* < .05, and primary statistical analysis was completed before November 18, 2022. All analyses were performed using the TwoSampleMR package (version 0.5.6) in R software (version 4.2.1).

## 3 RESULTS

### 3.1 Genetic correlations between COVID‐19 and MG

As shown in Table 1, our LDSC analysis revealed no genetic associations between COVID‐19 susceptibility and MG (*Rg* = .045, *p* = .607), early‐onset MG (*Rg* = −.207, *p* = .135), or late‐onset MG (*Rg* = .131, *p* = .147). Furthermore, there was no evidence supporting genetic associations between COVID‐19 hospitalization and MG (*Rg* = .094, *p* = .258), early‐onset MG (*Rg* = .027, *p* = .818), or late‐onset MG (*Rg* = .092, *p* = .259). Similarly, we found no genetic associations between COVID‐19 severity and MG (*Rg* = .055, *p* = .543), early‐onset MG (*Rg* = −.064, *p* = .586), or late‐onset MG (*Rg* = .092, *p* = .324).

**TABLE 1:** The genetic correlations between coronavirus disease 2019 (COVID‐19) phenotypes and myasthenia gravis (MG).

Exposure	Outcome	*Rg*	SE	*p*‐Value
COVID‐19 susceptibility	MG	.045	.087	.607
	Early‐onset MG	−.207	.139	.135
	Late‐onset MG	.131	.090	.147
COVID‐19 hospitalization	MG	.094	.083	.258
	Early‐onset MG	.027	.116	.818
	Late‐onset MG	.092	.081	.259
COVID‐19 severity	MG	.055	.091	.543
	Early‐onset MG	−.064	.118	.586
	Late‐onset MG	.092	.093	.324

*Note*: *Rg*, genetic correlation.

### 3.2 Causal relationships between COVID‐19 and MG

**FIGURE 1:**
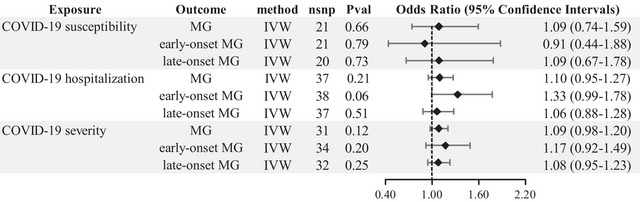
IVW results of COVID‑19 phenotypes on the risk of MG. IVW, inverse‐variance weighted method; MG, myasthenia gravis.

Our MR analysis revealed no evidence to support a causal relationship between genetically determined COVID‐19 susceptibility (IVW: OR: 1.09, 95% CI: .74–1.59, *p*: .66), hospitalization (IVW: OR: 1.10, 95% CI: .95–1.27, *p*: .21), and severity (IVW: OR: 1.09, 95% CI: .98–1.20, *p*: .12) with MG (Figure 1, Table S1). Furthermore, no causal relationship was found between COVID‐19 susceptibility (IVW: OR: .91, 95% CI: .44–1.88, *p*: .79), hospitalization (IVW: OR: 1.33, 95% CI: .99–1.78, *p*: .06), and severity (IVW: OR: 1.17, 95% CI: .92–1.49, *p*: .20) with early‐onset MG (Figure 1, Table S1). Similarly, no causal relationship was found between COVID‐19 susceptibility (IVW: OR: 1.09, 95% CI: .67–1.78, *p*: .73), hospitalization (IVW: OR: 1.06, 95% CI: .88–1.28, *p*: .51), and severity (IVW: OR: 1.08, 95% CI: .95–1.23, *p*: .25) with late‐onset MG (Figure 1, Table S1). The *F*‐statistics for all genetic variants were greater than 10.

Sensitivity analysis showed that Cochran's *Q* test did not detect any horizontal pleiotropy (*p* > .05, Table 2). No horizontal pleiotropy was detected by the MR‐Egger intercept test. The MR‐PRESSO analysis did not identify any outliers (*p* > .05, Table 2). In addition, we used the PhenoScanner online tool to manually search for IVs related to age, thymoma, and autoimmune diseases. Our MR estimates remained consistent with the original results after removing the SNPs associated with these confounding factors (Table S2). Additionally, we assessed the proportion of variance explained by the included IVs representing the exposure traits, which ranged from 1.8% to 26.0% for different COVID‐19 phenotypes. Importantly, all of our MR estimates had a statistical power greater than .8, indicating sufficient power for the analysis (Table S3).

**TABLE 2:** Sensitivity analysis of coronavirus disease 2019 (COVID‐19) phenotypes on myasthenia gravis (MG).

MR analysis		Heterogeneity		Horizontal pleiotropy			
Exposure	Outcome	Cochran's *Q*	*p* Value	Egger intercept	*p* Value	MR‐PRESSO *p* value	Outlier
COVID‐19 susceptibility	MG	22.31	.32	.03	.11	.37	0
	Early‐onset MG	21.36	.38	.00	.90	.43	0
	Late‐onset MG	24.73	.17	.04	.12	.21	0
COVID‐19 hospitalization	MG	35.66	.48	.01	.54	.52	0
	Early‐onset MG	38.53	.40	02	.43	.44	0
	Late‐onset MG	41.79	.23	.01	.52	.27	0
COVID‐19 severity	MG	29.70	.48	.02	.10	.51	0
	Early‐onset MG	48.14	.04	.03	.36	.06	0
	Late‐onset MG	36.41	.23	.02	.21	.26	0

## 4 DISCUSSION

To our knowledge, this study is the first to evaluate the genetic association and potential causal relationship between COVID‐19 and MG. Using LDSC and MR analysis, no significant genetic or causal association was observed between COVID‐19 and MG phenotypes. The extensive sensitivity analysis performed confirmed the primary findings, thereby enhancing the stability and consistency of the MR analysis.

By employing cross‐trait LDSC analysis with two different GWAS datasets at the summary level, this study effectively addresses the issue of sample overlap and allows for the estimation of global correlation (Bulik‐Sullivan et al., 2015; Yang et al., 2022). In the present MR studies, genetic IVs that show strong associations with both COVID‐19 and MG are used as reliable proxies for exposure and outcome. This approach serves to reduce the effects of confounding and reverse causation, thereby strengthening the causal inference between exposure and outcome (Burgess et al., 2013; Yuan et al., 2021).

In the absence of large‐scale randomized controlled trials, the reanalysis of GWAS data at the summary level using LDSC and MR methods is undoubtedly a valuable avenue of investigation. As preliminary investigations, these approaches aim to shed light on the association and causality between COVID‐19 and MG, thus filling an important gap in the field. Furthermore, by exploring the association between COVID‐19 and MG, it will be possible to identify common pathogenic mechanisms, which in turn may provide new insights into potential molecular mechanisms and therapeutic development (Patrick et al., 2023). Although current research efforts have not uncovered significant associations between the two diseases, these efforts remain critical to advancing our understanding of these complex diseases.

Previous studies have confirmed that SARS‐CoV‐2 can enter the nervous system and induce a cytokine storm and immune dysregulation (Gu et al., 2005; Ismail & Salama, 2022; Kuhlmann et al., 2023), which is a risk factor for several neurological disorders (Aghagoli et al., 2021; Harapan & Yoo, 2021). There is limited evidence for a causal relationship between COVID‐19 and immune disorders such as multiple sclerosis and hypothyroidism (Baranova et al., 2023; Li et al., 2022; Zhang et al., 2022). Researchers have speculated that potential molecular mimicry between acetylcholine receptors and SARS‐CoV‐2 proteins leads to post‐infection MG (Assini et al., 2021; Tereshko et al., 2023), but there appears to be no apparent structural match between subunits of acetylcholine receptors and SARS‐CoV‐2 proteins (Huber et al., 2020; Muhammed et al., 2021). Although these hypotheses attempt to explain the causal relationship between COVID‐19 and MG, they are supported more by chance in our study. We cannot rule out bias in the MR analysis, but extensive sensitivity analyses make this scenario highly unlikely. Therefore, we tend to believe that the reported cases may be due to COVID‐19 infection or vaccination unmasking latent MG. Specifically, the innate immune system is stimulated by COVID‐19‐related stimuli, releasing preexisting self‐antigens of acetylcholine receptors (Chavez & Pougnier, 2021). However, more research focused on these topics is needed in the future.

Emerging evidence suggests a possible association between COVID‐19 and MG, but research results remain controversial. Some case reports or series have shown MG following COVID‐19 vaccination or infection (Chavez & Pougnier, 2021; Fanella et al., 2022; Restivo et al., 2020; Sriwastava et al., 2021; Muralidhar Reddy et al., 2021), suggesting that the onset of MG has a clear temporal sequence after COVID‐19. The prevalence of case reports in the literature naturally leads to speculation about a possible association, although the possibility of coincidence cannot be excluded. Recent cohort studies have suggested an increased risk of MG associated with SARS‐CoV‐2 infection. A matched study of 4411 MG patients found an absolute mortality rate of 14.6% within 30 days, higher than the general population (8.5%) (Alcantara et al., 2023). Roy et al. (2021) showed that MG patients with SARS‐CoV‐2 infection had a higher risk of hospitalization (OR: 3.0; 95% CI: 2.4–3.8) and death (OR: 4.3; 95% CI: 2.9–6.4), consistent with several other studies (Doron et al., 2022; Peric et al., 2023; Roy et al., 2021). However, other studies have not found an increased risk of MG with COVID‐19. A 10‐year real‐world data study by Wartmann et al. (2023) showed lower MG incidence and hospitalization rates in 2020 compared to 2011–2022, although this may be due to the impact of the COVID‐19 pandemic. An Italian cohort study of 162 MG patients found that COVID‐19 had a minimal effect on the course of MG (Businaro et al., 2021). A retrospective observational study of 83 MG patients with COVID‐19 infection evaluated clinical features and outcomes and showed that most MG patients with COVID‐19 did not require hospitalization or experience exacerbation of MG. However, it is important to note that this study lacked a control group from the general population, so the results must be interpreted with caution (Karimi et al., 2022).

Currently, the results from traditional observational studies are inconsistent. Some evidence does not elucidate whether the higher hospitalization and mortality rates are due to multi‐organ failure, COVID‐19, exacerbation of autoimmune dysfunction, or exacerbated MG treatments (Galassi, 2022). It cannot be completely ruled out that MG may lead to severe COVID‐19 and subsequently increase the risk of hospitalization and mortality (Moura et al., 2022). It is important to note that certain studies have included MG populations comprising both AchR and MuSK antibody‐positive patients (Doron et al., 2022; Peric et al., 2023; Thomas et al., 2023). It has been confirmed that MG patients with MuSK or AchR antibodies exhibit distinct clinical features and biochemical markers (Gilhus et al., 2019; Huang et al., 2023). In our analysis, we solely focused on AchR antibody‐positive patients, which may contribute to the inconsistency observed when comparing our results to certain findings from observational studies (Alcantara et al., 2023; Roy et al., 2021).

In our analysis, the results from LDSC and MR studies did not reach statistical significance. In other words, we did not find a positive association between COVID‐19 and increased risk of MG, which is consistent with some observational studies (Businaro et al., 2021; Wartmann et al., 2023). This may also shed light on the ongoing safety controversy surrounding COVID‐19 vaccination in MG patients. Considering COVID‐19 vaccines as attenuated versions of the SARS‐CoV‐2 virus, we can conclude that COVID‐19 vaccination in MG patients is likely to be safe from a genetic point of view. Our reasoning aligns with several cohort studies (Ishizuchi et al., 2022; Lupica et al., 2022; Ruan et al., 2021; Trinchillo et al., 2023; Zheng et al., 2023). A recent cohort study investigating 113 fully vaccinated MG patients found that the COVID‐19 vaccine was safe for these individuals (Zheng et al., 2023). Ruan et al. (2021) demonstrated that vaccination, even with different types of vaccines, was safe in MG patients, and a few individuals who experienced brief worsening within 1 month after vaccination did not require rescue treatment. Ishizuchi et al. (2022) examined 294 vaccinated MG patients and did not observe any critical events, with only 1% of patients experiencing worsening, but without the need for mechanical ventilation or admission to the intensive care unit. A meta‐analysis conducted by Zheng et al. indicated that although a small proportion of vaccinated MG patients experienced transient worsening, vaccination was generally safe for most MG patients (Trinchillo et al., 2023). These findings are also supported by several other studies (Alcantara et al., 2023; Farina et al., 2022; Lupica et al., 2022; Shah et al., 2022).

Considering the evidence from these observational studies and our analysis, MG patients show good safety and tolerability to SARS‐CoV‐2 vaccines, and vaccination can be considered to prevent severe and life‐threatening complications.

Our study has several strengths. First, we comprehensively evaluated the relationship between COVID‐19 and MG using publicly available GWAS data. Second, our MR analysis minimized the confounding biases and potential for reverse causation. Third, extensive sensitivity analyses strengthened the stability and consistency of our MR estimates. However, there are also limitations to our study. First, our genetic data mainly came from European populations, so our results may not be easily extrapolated to other ethnic groups. Second, LDSC and MR analyses only use summary‐level data from GWAS, which precludes further genetic analyses at the individual level, such as assessing the impact of medications. Third, although we did not find any association between COVID‐19 and MG phenotypes, including all MG, early‐onset MG, and late‐onset MG, it should be noted that MG can also be classified into ocular and generalized MG. In addition, a small percentage of MG patients carry MuSK and/or LRP4 antibodies rather than AchR antibodies. The association between COVID phenotypes and these MG subgroups is still unknown. Validation of the current analysis requires GWAS research focusing on MuSK and/or LRP4 antibodies. In addition, our LDSC and MR studies are preliminary and caution should be used in interpreting the present results. More robust evidence, such as cohort studies, is needed to evaluate the association between COVID and MG.

In conclusion, our study found no evidence of genetic association or causal relationship between COVID‐19 susceptibility, hospitalization, and severity and MG. This suggests that COVID‐19 vaccination may be safe in MG patients. Our study provides new evidence to understand the impact of COVID‐19 on neurological immune disorders. Future GWAS data are needed to evaluate the association between COVID‐19 phenotypes and MG and its subgroups.

## AUTHOR CONTRIBUTIONS

Hongyu Zhou conceived and designed the overall research. Dongren Sun and Liangdan Tu analyzed the data and wrote and revised the manuscript. All authors were involved in the collection and monitoring of the data. All authors reviewed and agreed to the submitted version.

## CONFLICT OF INTEREST STATEMENT

The authors declare no conflicts of interest.

### PEER REVIEW

The peer review history for this article is available at https://publons.com/publon/10.1002/brb3.3239.

## Supporting information


**Table S1** Estimation of MR by MR‐Egger, weighted median, simple mode, and weighted mode.

**Table S2** MR reanalysis after the removal of confounding factors by PhenoScanner online tool.

**Table S3**. Variance and power of COVID‐19 phenotypes and MG phenotypes.

## Data Availability

All results generated from this study were derived from publicly available GWAS summary data.
